# Evaluation of ultrasound lung comets by hand-held echocardiography

**DOI:** 10.1186/1476-7120-4-34

**Published:** 2006-08-31

**Authors:** G Bedetti, L Gargani, A Corbisiero, F Frassi, E Poggianti, G Mottola

**Affiliations:** 1 Imola Hospital, Imola, Italy; 2 CNR, Institute of Clinical Physiology, Pisa, Italy; 3 Montevergine Clinic, Mercogliano, Italy

## Abstract

**Background:**

Ultrasound lung comets (ULCs) are a clinically useful sign of extravascular lung water. They require very limited technology (2 D-echo), and a short learning curve.

The aim of the present study is to compare ULCs information obtained by experienced echocardiologists using a full feature echocardiographic platform and by inexperienced sonographers using a hand-held echocardiography system.

**Methods:**

20 consecutive in-hospital patients underwent, within 15' and in random order, 2 ultrasound examinations for ULCs by 2 observers with different specific expertise and different technology: 1) "high-tech veteran": ULCs assessment with full feature echocardiographic platform (HP Sonos 7500 Philips Medical Systems, Andover, MA, USA) by a trained echocardiologist, with ≥2 years expertise in ULCs assessment and accredited by the European Association of Echocardiography; 2) and a "low-tech beginner": ULCs assessment with hand-held echocardiography (Optigo; Philips, Andover, MA) by an echocardiographer with very limited (30') dedicated training on ULCs assessment.

In each patient, ULC score was obtained by summing the number of comets from each of the scanning spaces in the anterior right and left hemithorax, from the second to the fifth intercostal space.

**Results:**

There was a significant, tight correlation (r = .958, p < 0.001) between the 2 observations in the same patient by "high-tech veteran" and "low-tech beginner".

**Conclusion:**

ULCs are equally reliable in the hands of highly experienced echocardiologists using full feature echocardiographic platforms and in the hands of absolute beginners with miniaturized, compact, and battery-equipped echocardiographic systems. From the technological and expertise viewpoint, ULCs are the "kindergarten" of echocardiography, ideally suited for bedside evaluation of patients with both known or suspected heart failure.

## Background

The interstitial pulmonary oedema is a key parameter in the management of patients with chronic heart failure and an early warning sign of impending acute heart failure [1]. The objective diagnosis is traditionally based on chest radiographic findings which, when performed at the bedside, may be difficult to interpret, and may have weak correlations with extravascular lung water [2,3]. The lung is considered poorly accessible using ultrasound since air prevents the progression of the ultrasound beam with production of reverberation artefacts under the lung surface [4]. The "comet-tail image" is an echographic image detectable at bedside with ultrasound probes positioned over the chest [5]. This image consists of multiple comet tails fanning out from the lung surface originating from water-thickened interlobular septa. Functionally, they are a sign of distress of the alveolar-capillary membrane, often associated with reduced ejection fraction and increased pulmonary wedge pressure and they probably represent an ultrasonic equivalent of radiologic Kerley B-lines [6]. These features make ultrasound lung comets (ULCs) an appealing simple clinically useful sign for detecting and quantifying extravascular lung water [7] in patients with known or suspected heart failure.

ULCs require very limited technology (2 D-echo, without Doppler functions or second harmonic option) and a short learning curve. Recently, hand-held echocardiography with compact, low cost, miniaturized, battery-driven ultrasound imaging system has became available [8].

The aim of our study is to compare the number of ULCs obtained by an experienced echocardiologist using a full feature echocardiographic platform and inexperienced sonographer using hand-held echocardiography and to evaluate the learning curve, the feasibility and the time needed for the echo lung examination.

## Methods

### Patients population

Twenty consecutive in-hospital patients (age 66 ± 12 years; 18 men), admitted to the cardio-pulmonary department of the Institute of Clinical Physiology, CNR in Pisa, have been included in the study.

### Echocardiography study

All patients underwent, within 15' and in random order, 2 ultrasound examinations for specific ULCs imaging by 2 observers utilizing different technology and with different expertise: 1) "high-tech veteran": ULCs assessment with a full feature echocardiographic platform (HP Sonos 7500 Philips Medical Systems, Andover, MA, USA) by a trained echocardiologist, with ≥2 years expertise in ULCs assessment and accredited by European Association of Echocardiography; 2) "low-tech beginner": ULCs assessment with hand-held echocardiography (Optigo; Philips, Andover, MA) by an echocardiographer with very limited (30') dedicated training on ULCs assessment.

### Chest ultrasound

**Figure 1:**
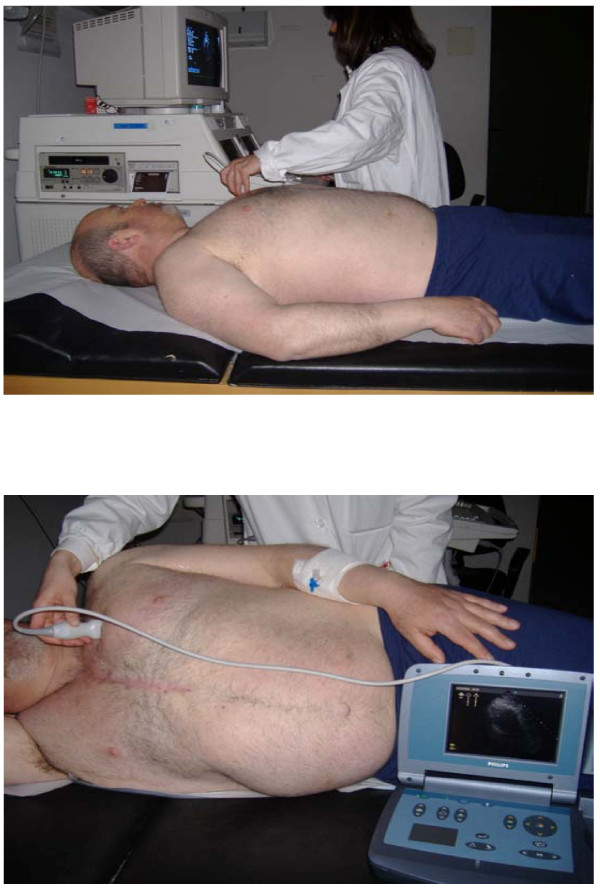
Echographic examinations are performed with patients in the supine or near-supine position.

The ULC was defined as a hyperechogenic, coherent bundle with narrow basis spreading from the transducer to the further border of the screen [9]. The ULC described here extends to the edge of the screen and arises only from the pleural line. The echographic examinations are performed with patients in the supine or near-supine position (Figure 1). Ultrasound scanning of the anterior and lateral chest is obtained on the right and left hemitorax, from the second to fourth (on the right side to the fifth) intercostal spaces, and from parasternal line to midaxillary line. In each intercostal space, the number of ULCs was recorded at the parasternal, midclavear, anterior and middle axillary lines [7]. The sum of the ULCs yielded a score denoting the extent of the extravascular fluid of the lung. Zero was defined as a complete absence of ULCs on the investigated area [9]. Observers were unaware of one another's results and ULCs measurements were independent of one another.

### Statistics

Data are expressed as mean ± 1 standard deviation for continuous variables. The correlation between the measurements of the two observers was analyzed by two-tailed Pearson bivariate correlation. The statistical analyses of the data has been performed with SPSS for Windows (version 13.0, SPSS Inc., Chicago, Illinois). A p value < 0.05 was considered to be significant.

## Results

**Figure 2:**
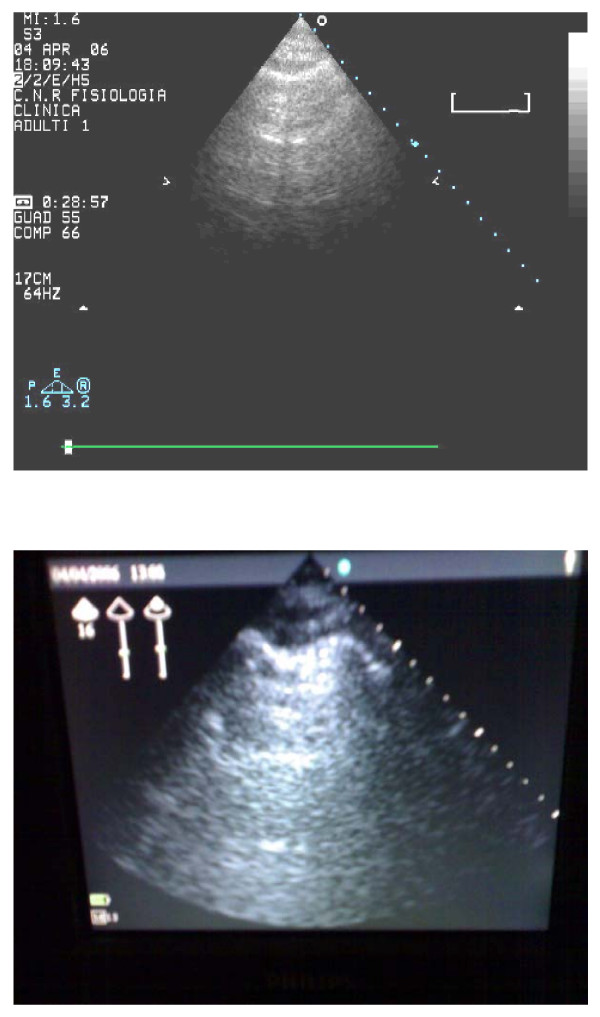
Example of patients without ULCs.

**Figure 3:**
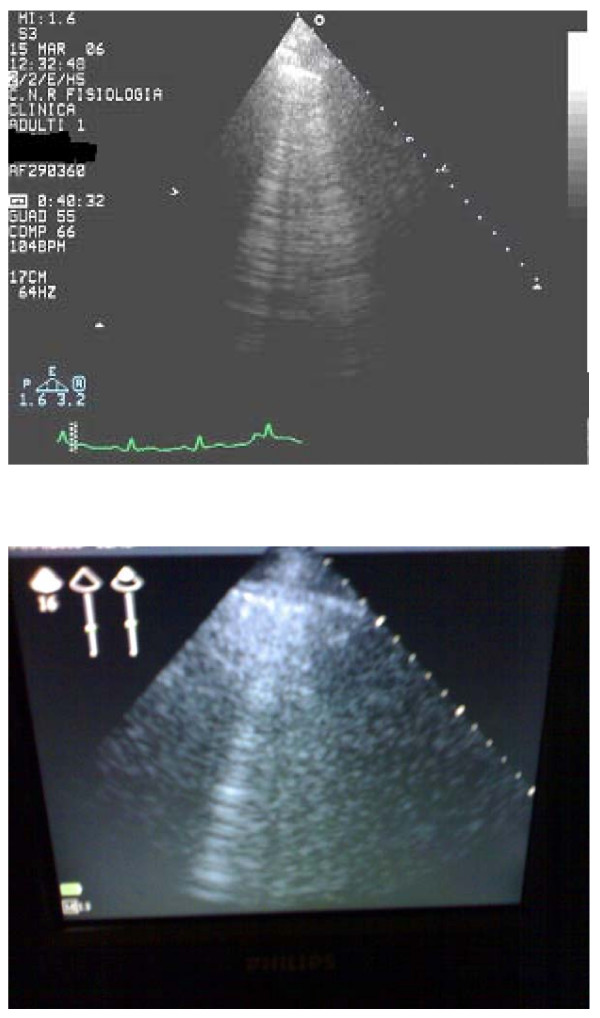
Example of patients with ULCs.

**Figure 4:**
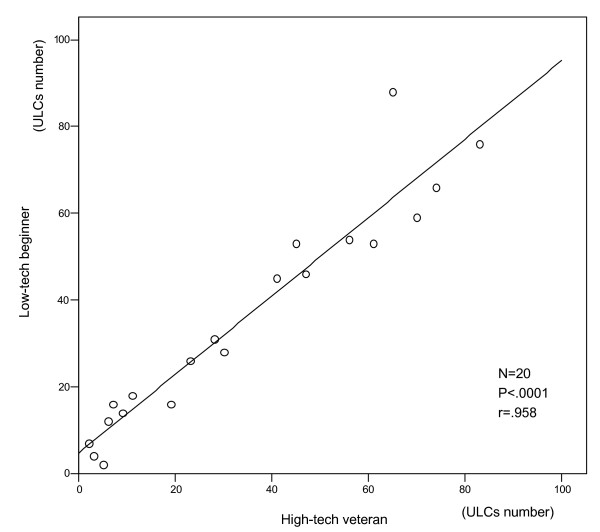
Graph showing the significant correlation between the 2 observations in the same patient by the "high-tech veteran" and the "low-tech beginner".

A total of 20 paired observations have been obtained for all the enrolled patients. The feasibility of the chest ultrasound examination for the diagnosis of ULCs was 100%. The time needed for the echo lung examination was <3 minutes in all patients. A significant (<5) number ULCs have been observed in 17 patients, while 3 patients showed a number of comets <5. The mean value of ULCs was 35.7 ± 25.30; and 34.2 ± 26.8 for the low tech beginner. Two examples of two representative patients with and without ULCs, are shown in Figures 2 and 3, respectively. The appearance and numbers was similar with the hand-held echocardiography (Fig. 2 and 3, lower panels) and with the full feature echocardiographic platform (Figure 2 and 3, upper panels). There was a tight and highly significant correlation between the 2 observations in the same patient by the "high-tech veteran" and the "low-tech beginner" (Figure 4).

## Discussion

ULCs are based on the principle that ultrasound is reflected by an interface between different acoustic impedance. In normal conditions, the ultrasound beam finds the lung air (ie, high impedance and no acoustic mismatch on its pathway through the chest) [6]. In the presence of extravascular lung water, the ultrasound beam finds sub-pleural interlobular septa thickened by oedema (ie, a low impedance structure surrounded by air and with a high acoustic mismatch). The reflection of the beam creates a phenomenon of reverberation. The result is a wedge – shaped signal with a narrow origin in the near field of the image [9]. We have found that ULCs are equally reliable in the hands of highly experienced echocardiologist using expensive and complex technology and in the hands of absolute beginners with hand-held echocardiography. ULCs are easy both to obtain and to measure (learning curve of <10 examinations, 30 minutes) and fast to perform (<3 minutes), require very limited technology, even without a second harmonic or Doppler and are not restricted by cardiac acoustic window limitations or patient decubitus. The feasibility was 100%.

### Clinical implications

Pulmonary congestion resulting from elevated left atrial and left ventricular filling pressures appears before the onset of patient symptoms, and may provide an early warning of impending heart failure. The possibility that sufficiently accurate techniques could be used to detect pulmonary oedema even before it becomes clinically apparent "is so inherently attractive that the effort to develop and validate such techniques still continues" [10].

The standard chest radiograph remains the best screening test for the detection of pulmonary oedema, but it requires radiology facilities, specific reading expertise, it uses ionising energy, and poses a significant logistic burden. ULCs assessment provides an appealing simple, non-radiologic and low cost bedside alternative to available methods and it appears to be reasonably well correlated with extra-vascular lung water assessed by chest radiography [7] and other highly complex methods such as computerized tomography and thermodiluition techniques [11]. It is theoretically appealing for detecting and quantifying extravascular lung water, a key parameter in the serial evaluation of the cardiologic patient with heart failure [13]. It seems attractive for complement conventional Doppler-echocardiography in the fast evaluation of patients with known or suspected heart failure and dyspnoea as a presenting symptom in the emergency department (for the differential diagnosis of dyspnoea), in-hospital management (for serial evaluations in the same patient and for tailoring diuretic therapy), home care (with hand-held echocardiography), and stress echocardiography lab (as a sign of acute pulmonary congestion during stress) [14].

## Conclusion

Since the qualitative nature of the interpretation is the recognized Achilles hell of echocardiographic techniques [15,16], it is especially reassuring that both the presence and extent of ULCs can be obtained with optimal precision, with very limited training, and with very basic and unsophisticated technology. From the technological and expertise viewpoint, ULCs are the "kindergarten" of echocardiography, ideally suited for bedside evaluation of patients with both known or suspected heart failure.

## Abbreviations

ULCs = Ultrasound lung comets

## Competing interests

The author(s) declare that they have no competing interests.

## Authors' contributions

LG, GB and EP performed all of the ULCs assessments. AC and FF performed all the statistical analysis of the study. GM participated in the study design and coordination an helped to draft the manuscript. All authors read and approved the final manuscript.
